# Decision criteria for replacement of fillings: a retrospective study

**DOI:** 10.1002/cre2.30

**Published:** 2016-07-04

**Authors:** J. Kirsch, J. Tchorz, E. Hellwig, T.T. Tauböck, T. Attin, C. Hannig

**Affiliations:** ^1^ Clinic of Operative Dentistry, Medical Faculty Carl Gustav Carus Dresden University of Technology D‐01307 Dresden Germany; ^2^ Department of Operative Dentistry and Periodontology University of Freiburg D‐79106 Freiburg Germany; ^3^ Centre for Operative Dentistry and Periodontology, University of Dental Medicine and Oral Health Danube Private University (DPU) AT‐3500 Krems an der Donau Austria; ^4^ Clinic of Preventive Dentistry, Periodontology and Cariology University of Zürich CH‐8028 Zürich Switzerland

**Keywords:** amalgam, composite, exchange of restorations, residual caries, secondary caries

## Abstract

The exchange of restorations goes along with the loss of healthy tooth structure. Therefore, it is important to investigate helpful decision criteria for the replacement of fillings. Five hundred forty‐four filling replacements were evaluated retrospectively. Thereby, different clinical parameters were correlated with the clinical finding of caries directly after removal of the existing filling. The parameters checked for correlations were amalgam and composite, age, and size of the filling, morphology, condition of the filling, type of caries, oral hygiene, anamnesis of the respective tooth, and the decisive factor to replace the restoration. Statistical evaluation was performed by chi‐squared‐test (*P* < 0,05) and by regression analysis (Power: 80%). A percentage of 69.8% of all cavities showed softened dentin if exploring with the probe after the removal of the restoration, 7.6% were stainable with caries detector, and 22.6% of the cavities were caries free. Significant indicators for a carious lesion were high age of restoration, imperfections at the margin of the filling, a positive pain sensation in correlation with composite fillings, and multi‐surface amalgam fillings. On suspicion of caries, the following decision criteria should encourage the dentist to remove a filling: High age of the filling, imperfections at the margin of the filling, especially fillings with marginal cracks, visible secondary caries, a positive pain sensation in composite filled teeth, and multi‐surface amalgam fillings. Filling removals only performed due to the patient's desire for removal should be critically regarded, as most of these fillings are caries free.

## Introduction

Dentists spend much time replacing deficient restorations (Mjör and Ryge 1981; Elderton and Davies 1984; Maryniuk 1984; Maryniuk and Kaplan 1986; Burke et al. 1999; Forss and Widström 2004; Setcos et al. 2004; Fernandez et al. 2011). This takes up a larger part than the filling of primary carious lesions and is very cost intensive for patients and the health system (Paterson et al. 1995). Furthermore, replacement of fillings always goes along with loss of dental hard tissue. Reasons for the breakdown of fillings are multiple. They range from defective margins of the fillings (Mjör and Ryge 1981; Braga et al. 2007), fractures, or secondary caries up to the total loss of a restoration (Forss and Widström 2004; Da Rosa Rodolpho et al. 2011). Other possible reasons are periodontal irritations, treatment of primary carious lesions at the restrictive tooth, washed‐out fillings, and esthetic aspects particularly at the anterior teeth. According to current studies, the primary reason is secondary caries followed closely by fracture of the tooth (especially in amalgam filled teeth) or the restoration itself (especially in composite filled teeth) (Mjör and Gordan 2002; Forss and Widström 2004; Opdam et al. 2010; Demarco et al. 2012). The life span of a restoration depends on the material used. Composite showed over years typical problems like fatigue shrinkage, higher wear rates, defective contact points leading to food impaction as well as insufficiently converted composite at the bottom of the cavity (De Moor and Delme 2008). Nowadays, the development of marginal defects (i.e., gap between the filling and the tooth) with secondary decay, fractures, and discolorations is the main problems with this material (Manhart 2004). Reasons for the replacement of defective amalgam restorations are secondary decay, marginal defects, and inadequate integrity (Hickel and Manhart 2001; Moncada et al. 2008) as well as partial or complete cusp fracture next to amalgam fillings or involving the amalgam restoration (Cehreli et al. 2010; Özcan et al. 2010; Blum et al. 2011). Opdam et al. (2010) evaluated the annual failure rate and showed that composite and amalgam restorations come up with equal results. Beside the material, size of the cavity is also very important for longevity of a restoration. The bigger the cavity – the shorter is the average life span of the restoration. This might be directly related with the susceptibility for fractures (Van Nieuwenhuysen et al. 2003; Opdam et al. 2007; Demarco et al. 2012). The caries risk of the individual patient is an additional parameter, which has to be recognized. Patients with high‐risk levels show much more failures of fillings than patients with a low caries level (Opdam et al. 2010). Traditionally, the replacement of the restoration is the first choice to treat insufficient fillings, but in some cases, repair of the existing restoration is a valuable and valid alternative (Mjör 1993; Foitzik and Attin 2004). It is essential that a repair is strictly limited to fillings with imperfections at the margin of the filling, where a secondary or residual caries underneath the filling can be excluded. The exchange of defective restorations always goes along with the loss of healthy tooth structure. Therefore, it is important to adapt the indications for filling replacements. Yet, there is a lack of effective parameters, so that decisions for removal of fillings are more influenced by individual clinical experience rather than by evidence, subjective reasons (Noack and Treige 1994), and local practice patterns (Drake et al. 1990; Elderton 1990; Burke et al. 1999; Deligeorgi et al. 2000; Sharif et al. 2014a). The aim of this study was to investigate useful decision criteria for the replacement of fillings. Directly after the removal of the existing filling, different clinical parameters were correlated with the clinical status of decay.

## Methods

This study was reviewed and approved by the ethics committee of the University of Freiburg and the ethics committee of the University of Zürich. A number of 544 filling replacements were evaluated in this study. The study was conducted at the university hospital in Freiburg/Breisgau, Germany, and the university hospital in Zürich/Switzerland from 2009 to 2011. A number of 394 subjects were examined in Freiburg/Breisgau, and 150 subjects participated in Zürich. The evaluation was accompanying the daily dental routine and was made anonymously. The filling replacements, if necessary, were performed in the dental students' class in both universities. A lecture and a practical training informed all of the participating students and instructors about the principals of the study and the examination form. The examination form was thereby conducted via multiple choice, and it was completed for every replaced restoration. Before replacing the filling, the clinical relevance of each filling removal was checked by the students' supervisors. The decision to replace a restoration was therefore made irrespective of the study. The average age of the patients was 48 years  ± 14 years (minimum: 24 years, maximum: 85 years): 48.1% of the patients belonged to the age cohort 41–60 years. The results showed 20.3% anterior teeth restorations and 79.7% posterior teeth restorations.

### Study design

After the removal of the old filling, different clinical parameters were correlated with the clinical status of decay. The main differentiations of the clinical findings of caries were (a) caries free cavities, (b) caries detector stainable (Kuraray, Okayama; Japan) cavities, and (c) cavities with softened dentin. The main correlating factors were material, age, and size of the filling. Only amalgam and composite fillings were included to be in good accordance with the study of Hannig et al. (2009). Other parameters recorded were morphology of reconstruction, condition of the filling, type of caries (secondary or residual caries), oral hygiene, anamnesis of the respective tooth, and the main decisive criterion to replace the restoration. The evaluation was recorded by using an examination form.

### General parameters

General hygiene (good/average/poor) and DMFT (decayed, missing or filled teeth index) was determined. The sulcus bleeding index (SBI) and the proximal plaque index (API) (Löe and Silness 1963; Silness and Löe 1964) were both recorded in a modified application based on Lange (1986).

### Other parameters

Position, age, material, and extension of the filling were gathered. The anamnesis form asked for the presumable age of the filling, sensitivity to pain, temperature sensation, sensitivity to sweetness, sharp edges, occlusal pain, loosened or fractured fillings and food impaction. Presence of carious dentin at the margins of the restoration was checked by clinical examination. In addition, the condition and the morphology of the filling were recorded. If available, existing X‐rays were also evaluated. Thereby, bitewing, panoramic (OPG), and periapical X‐rays were considered by the examiner. No extra X‐rays were made in the context of the study to avoid additional radiation exposure.

After the removal of the filling, the cavity was evaluated by the examiner with the help of magnifying glasses. The cavities were recognized as caries free, stainable with caries detector, and softened dentin. The study only examined decay at the floor of the cavity after removal of the filling. At the bottom of the examination form, the examiner had to define the most relevant criterion for the removal of the filling.

### Statistics

Statistical evaluation was performed by Pearson's chi‐squared test (*P* < 0.05). The power of the study is 80% (software: nQuery 7.0). In addition, a regression analysis was performed. The software used was spss statistics 21.0 (IBM, Ehningen, Germany).

## Results

**Figure 1:**
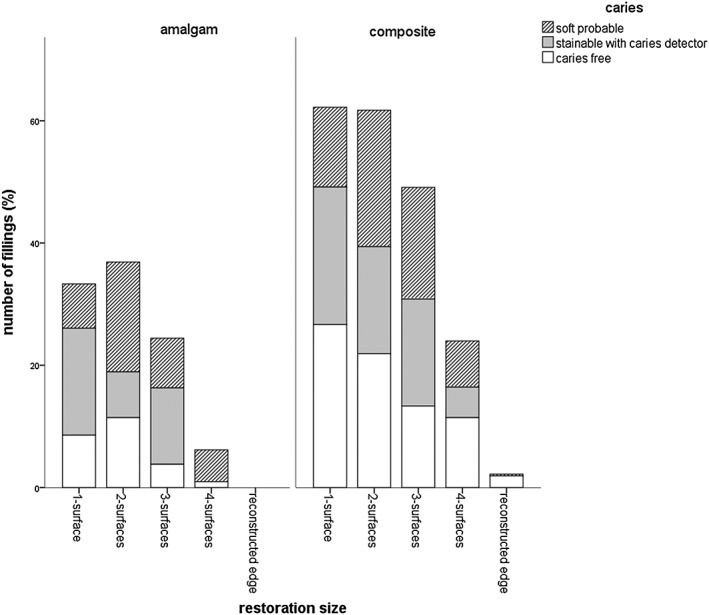
Distribution of restoration size and the clinical finding of secondary caries for amalgam and composite restorations.

**Figure 2:**
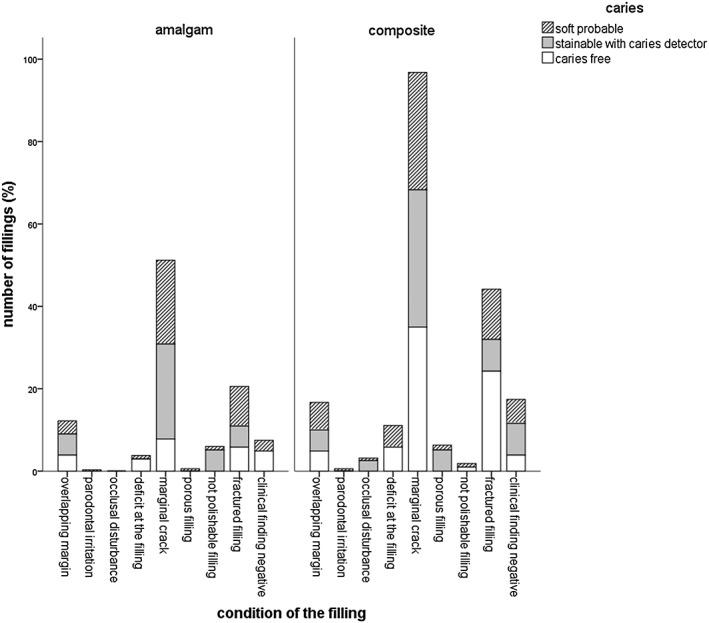
Distribution of the condition of the filling as well as the clinical finding of secondary caries for amalgam and composite restorations.

**Figure 3:**
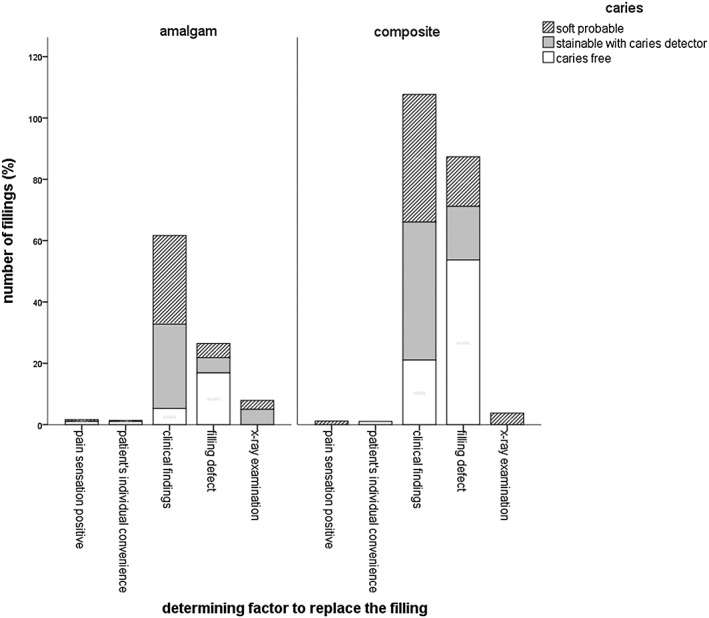
Distribution of the decisive factor to replace the filling and the clinical finding of secondary caries for amalgam and composite restorations.

**Figure 4:**
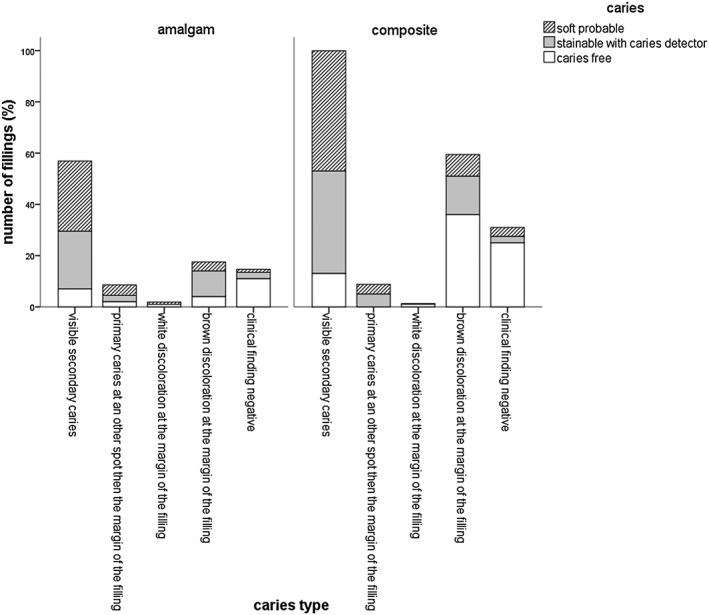
Distribution of the caries type of the treated tooth as well as the clinical finding of secondary caries for amalgam and composite restorations.

The majority of the participating patients were between 41 and 60 years old (48%). A percentage of 69.8% of the cavities showed softened dentin when explored with the probe, 7.6% were only stainable with caries detector, and 22.6% were caries free; 64.5% of the examined fillings were composite fillings, and 35.4% were amalgam fillings. The distribution of the fillings' localization was balanced (distal: 28.1%, mesial: 22.6%, distal and mesial: 20%, no proximal surface: 28.3%). Altogether, the average age of the replaced amalgam fillings was 15.3 ± 6.6 years and 9.4 ± 5.4 years for composite fillings, 30% of all restorations survived 10–20 years. In composite fillings and 41.7% in amalgam fillings, 58.3% of the detected secondary caries was found. Thereby, multi‐surface filled amalgam fillings showed a correlation between the size of the filling and the finding of secondary caries (Fig. 1). The highest rate of secondary or residual caries was found in two‐surface sized fillings (48.7% softened dentin + 20.3% caries detector stainable cavities), three‐surface sized fillings (33.2% + 51.2% caries detector stainable cavities), and four‐surface sized fillings (84.6% softened dentin). Composite fillings showed no correlation between the size and the caries susceptibility. The mean API was 47.1 ± 25.5%, and the mean SBI was 30.6 ± 24.6%. There was no correlation between caries finding and the API, SBI, and DMFT. The DMFT showed the following values: decayed 4.3 ± 4.6, missing 3 ± 3.3, filled 12.7 ± 5.3, and teeth 25.7 ± 4. The statistical evaluation showed a significant correlation between the filling's condition as well as the clinical finding of secondary or residual caries for the examined amalgam fillings (Fig. 2). If the following conditions were met, there was a significant chance for secondary or residual caries: in amalgam fillings with an overlapping margin, there was a 68.2% (26.2% softened dentin and 42% caries detector stainable cavities) chance for decay. Fractured amalgam fillings showed in 71.6% (46.7% softened dentin and 24–96% caries detector stainable cavities) a detectable caries at the bottom of the cavity. The prevalence of softened dentin in amalgam fillings with marginal cracks was 85.8%. Out of all amalgam fillings, 60 fillings had a marginal crack; 113 marginal cracks were found in all of the composite fillings. Interestingly, these composite fillings had a 63.9% probability to detect caries (softened dentin or caries detector stainable). The statistical analysis of the decisive key factor to replace the filling, the filling material, and the caries detectability showed a significant correlation (Fig. 3). In suspicion on secondary caries, dental diagnostic imaging was performed. X‐ray examination detected caries in 100% of the suspected cases for amalgam and composite fillings. In comparison with clinical examination, the caries detection rate was 91% in amalgam fillings and 80% in composite fillings; 2/3 of the fillings with marginal defects were caries free. A positive sensation of pain of teeth restored with composite indicated a carious lesion in 100% of the cases (Fig. 3); in an amalgam filled tooth, positive pain sensation just indicated carious dentin in the cavity in 35% of the cases. Another significant indication for caries was a visible secondary caries in amalgam and composite filled teeth (Fig. 4). Brown discolorations at the margin of the composite fillings indicated caries underneath the fillings in 39.4% of the cases.

None of the other criteria showed a significant correlation between the material and caries in the cavity; just tendencies could be recognized. The older the amalgam filling, the more caries was detectable. It is noteworthy that higher percentages for caries are starting at a presumed service life of 6 years in amalgam filled teeth. In composite filled teeth, the 50% rate for a possible clinical caries cavity is exceeded after 2 years of average service time. Anamnesis‐positive teeth (pain sensation, temperature sensation, sensitivity to sweetness, sharp edge, occlusal pain, fractured filling, food impaction, and no afflictions) had a high prevalence of caries (softened dentin and stainable with detector). A lower prevalence of caries was observed in teeth with a good reconstructed morphology, and every tooth surface less than average in reconstruction showed higher amounts of caries. This applied for both restorative materials. Upon closer inspection, the separated data from the two universities are in most parameters congruent.

## Discussion

This evaluation examined 544 cavities after the removal of a restoration. The evaluation in this study was carried out with an examination form. This examination form is a further development of the modified evaluation scheme of the California Dental Association from 1977, which was developed for quality assessment of fillings (Pieper 1990). It was prepared in such a way that the collection of the data was conducted after the clinical treatment (routine treatment). At that point, the clinical decision was already taken before the data were collected. With this procedure, the decision to replace a filling had not been distorted or influenced. Prior to the study performance, the participating students and dentists were calibrated, thereby detailed instructions and pretrial runs helped to avoid possible mistakes. This evaluation in the student dental class has advantages and disadvantages. On the one hand, the students have limited experiences in treating patients. Therefore, objective parameters are used for the assessment of restorations. On the other hand, their clinical decision is based on the established doctrine of their university. Besides, their supervisors at the dental school directly controlled every step in their treatment routine. At the end of the examination form, the examiner had to define the most relevant criterion for the removal of the filling. This criterion is really important, because it led the examiner to the removal of the restoration. Before, the examiner recorded the condition of the filling without any specific points of emphasis. Afterwards, the examiner decided on the most relevant criterion for the removal of the filling. Thereby, it has to be noted that the examiner mostly had to decide between a medical (condition of the filling and caries type) or an esthetic criterion (clinical findings, Fig. 3). Especially, clinically visible secondary caries and pain sensations of composite filled teeth led to the correct decision to replace the filling. Nevertheless, the treatment decision has to be considered critically because there are always different opinions about the same treatment situation (Merrett and Elderton 1984; Nuckles et al. 1991). It was established that the subjective impression of the examiner played an important role in the decision‐finding process. Marynuik (1984) postulated that the removal of a restoration depends on the individual clinical evaluation and clinical experience of the dentist. To verify the efficacy of the decision criteria, the results of both universities were evaluated separately from each other. It has been shown that the objective parameters the students were given in the evaluation form helped to decide a treatment situation. The resulting numbers were even congruent to the evaluation at the other university. In addition, it would be interesting to conduct the study in further countries. This would allow comparison of different approaches in the certain countries.

In 139 cases, the presumed age of the amalgam filling could be determined. Altogether, the average age of the replaced amalgam fillings in this study was 15.3 ± 6.6 years and 9.4 ± 5.4 years for composite fillings. Noticeable is the high longevity of amalgam restorations in comparison with composite. Different results were recorded by studies of Hickel and Manhart (2001), Hickel et al. (2005). They analyzed the longevity of restorations and observed a similar failure rate, if inserted correctly, between amalgam (0–7%) and composite (0–9%). The study of Heintze and Rousson (2012) screened 373 clinical studies. They postulated that the overall success rate of these filling materials is about 90% after 10 years and that there is no difference in longevity between the two filling materials.

In comparison with the present study, the study of Hannig et al. (2009) yielded different results in their findings of caries as seen in the following parentheses. A percentage of 69.8% (66.9%) of the cavities showed softened dentin when exploring with the probe, 7.6% (16.1%) were stainable with caries detector, and 22.6% (17%) were caries free. Nevertheless, it should be noted that the present study screened a higher number of filling removals than the study of Hannig et al. (2009) (544 filling removals vs. 317 filling removals). Besides, the present study showed no correlation between the size and the caries susceptibility of composite restorations. Two‐surface, three‐surface, and four‐surface sized amalgam fillings had significantly more caries underneath the fillings than one‐surface sized fillings and reconstructed edges (Fig. 1). In this context, the study of Opdam et al. concludes (Opdam et al. 2014) that larger composite restorations have a higher risk for failure too. Thereby, every extra surface included in a restoration increases this specific risk by 30–40%. Leak margins of fillings favor secondary caries and hypersensitivity in teeth (Hannig and Friedrichs 2001). The marginal integrity is a main problem of class‐II‐cavities. In this context, the present study also showed that out of all composite fillings, 113 fillings had a marginal crack; 60 marginal cracks were found in all of the amalgam fillings (Fig. 2). A percentage of 52.8% of the fillings with marginal cracks had a marginal crack size <0.4 mm, and 47.2% of the fillings with marginal cracks had a marginal crack size >0.4 mm. There was an 85.8% chance for amalgam and a 63.9% chance for composite restorations to detect caries (softened dentin or caries detector stainable).

In 1988, Weiland et al. (1988) already showed a correlation between the localization and the quality of a restoration. The level of difficulty is increased in fillings with a proximal part, and it is a predilection site for decay, especially at the posterior teeth. In the present study, 70.8% of all the removed fillings had a proximal part. Therefore, X‐ray examination is a decisive factor in the cariological and clinical findings (Fig. 3). The caries detection rate in combination with X‐rays is 70% higher than the examination without X‐rays (White et al. 1994; Foitzik and Attin 2004).

Marginal imperfections and overlapping filling margins are the main factor for proximal plaque accumulation (Hakkarainen and Ainamo 1980). In general, the oral hygiene reflects the complete hygiene of the patient and is an important indicator for secondary and residual caries (Hannig et al. 2009). The API in general is a very strict judgment of the oral hygiene. Therefore, the dentist should better get an overall impression of the patient's oral hygiene. This might be a reason why the present study could not show any correlation between caries under the removed filling and the API.

The surface morphology of the filling is also a good indicator for caries susceptibility, as the overall quality of a filling is directly related to the caries excavation and the accurate work of the dentist (Jahn and Binus 1980; Wöstmann and Lütke‐Notarp 1991). There was no definite significance and correlation between a good, average, poor and non‐reconstructed morphology and the presence of caries in this study. In contrast, Wöstmann and Lütke‐Notrap (1991) showed that underneath fillings with a good reconstructed morphology (evaluation of 1000 amalgam fillings) there is a 10 to 20 times less rate of decay than underneath fillings with a poor surface quality.

In the present study, 75% out of the caries free cavities were related to the filling material composite. From a purely cariological point of view, the filling removal was not justified, yet the patient's desire for a filling removal is an increasingly important factor. Especially, the esthetic of anterior teeth restorations plays an important role, particularly if these fillings are discolored. After filling removals only performed due to the patient's individual convenience, all examined cavities were caries free in this study (Fig. 3). Hickel and Klaiber (1992) showed that the esthetic appearance plays an important role of decision criterion for filling replacements beside size, localization, and proximal range of the filling. In our study, 60.5% of the composite fillings with brown discolorations at the margin were caries free. That means, if there are discolorations of the margins of composite fillings without any evidence of decay, the filling does not need to be removed completely. Söderholm and Roberts (1991) postulated that in this case, it is possible to repolish or to replace the outer layer of the restoration. However, in occlusal restorations, margin discoloration has shown to be an indicator for caries under the filling (Söderholm and Roberts 1991).

In some cases, repair offers an alternative for filling replacements, especially with respect to the conservation of healthy tooth structure and the pulp. Additionally, the financing needs of the patient can be eased, and the treatment time reduced (Kamann and Gängler 2000). Microleakage of saliva or sulcus fluid into gaps of insufficient restorations can lead to pulpal irritations or secondary decay. Under this aspect, replacement of a filling might be a reasonable decision instead of repair of the respective filling. However, repeated replacement leads to an increase in cavity size (Mjör 1998). Sharif et al. (2014a, 2014b) and Martin et al. (2013) came to the same conclusion. They evaluated amalgam restorations that were treated by repair or replacement. They suggest that repair treatment is as effective as total replacement of restorations with localized defects, reducing biological costs to the patient, and providing new tools to the clinician and that refinishing of a restoration is a useful treatment for localized anatomic form defects.

## Conclusion

Based on the present data, the following conclusions can be drawn. On suspicion of caries, the examiner should use the following criteria indicating caries under the restoration to replace an amalgam or composite filling: high age of the filling, imperfections at the margin of the filling, especially fillings with marginal cracks, clinically visible secondary caries, and a positive pain sensation in composite filled teeth. Furthermore, multi‐surface amalgam fillings should be checked carefully. Usually, brown marginal discolorations of composite fillings do not indicate caries under the filling. Filling removal conducted due to the patient's desire should be considered critically, as most of these fillings are caries free.

## Conflict of Interest

The authors declare that they have no conflict of interest.

## Source of Funding

There was no source of funding.
